# Spanish menstrual literacy and experiences of menstruation

**DOI:** 10.1186/s12905-023-02293-4

**Published:** 2023-04-04

**Authors:** Sara Sánchez López, Dani Jennifer Barrington, Rocio Poveda Bautista, Santiago Moll López

**Affiliations:** 1 Universitat Politècnica de València, Valencia, Spain; 2 University of Western Australia, Albany, Australia; 3 INGENIO (CSIC-UPV), Universitat Politècnica de València, Valencia, Spain; 4 DMA, Universitat Politècnica de València, Valencia, Spain

**Keywords:** Menstruation, Menstrual literacy, Information, Education, Taboo, Stigmatisation, Period poverty, Menstrual health, Menarche, Gender equality

## Abstract

There is growing recognition of the important role menstrual health plays in achieving health, education, and gender equity. Yet, stigmatisation and taboo remain present and negative emotions like fear and shame dominate the narrative when speaking about periods. This paper analyses how formal and informal menstrual education is received in Spain, to understand the role of menstrual health literacy in the way menstruation is experienced, and to identify what information would be useful to integrate into formal menstrual education. An online survey with more than 4000 participants (aged between 14 and 80, both people who will/do/have previously menstruate/d and those who do not menstruate) was conducted. Data was gathered using the digital platform Typeform, descriptive and inferential statistical analyses were performed with SPSS software and qualitative data was thematically analysed using Nvivo. Many participants declared not having received sufficient information on menstruation prior to menarche, particularly about how to physically manage it. Furthermore, negative emotions like shame, worry, and fear were recurrently reported to describe menarche; this has not changed between generations. Interestingly, we saw an increase in stress and sadness with an increase in perceived knowledge of the reproductive role of menstruation. We did observe a reduction in negative emotions when people who menstruate perceived they had sufficient information on how to manage their first bleeding. It is recommended that menstrual education beyond reproductive biology, particularly including how to physically manage periods, is integrated into school curricula. Menstrual education of everyone – including those who do not menstruate—can improve how periods are experienced in Spain.

**Supplementary Information:**

The online version contains supplementary material available at 10.1186/s12905-023-02293-4.

## Introduction

Approximately ten percent of the global population is experiencing menstruation at any given time [1]. Yet the negative impacts that stigmatisation, socio-cultural practices, and the cost of menstrual products have on the health and well-being of those who menstruate remain a reality [2–5].

Menstrual literacy is a person's knowledge of the menstrual cycle and how to care for their health and well-being when menstruating. Menstrual literacy is essential for the achievement of menstrual health, recognised as an integral part for the realization of human rights of all genders, the achievement of gender equality, and the Sustainable Development Goals [6]. Menstrual health literacy is crucial to tackle taboos, stigma, and discrimination related to the menstrual cycle to pave the way towards gender equality [7, 8] ​.

Menstrual literacy is low worldwide across geographic, economic, and cultural groups [9, 10]. A study in the UK reported that 14% of girls did not know what was happening, and 1 out of 4 girls did not feel they knew what to do, when they experienced their first period [2].

People who menstruate (PWM, including those who expect to, currently do, or have previously menstruate/d) often lack access to reliable information about periods [2, 3, 11]. Worldwide, PWM learn that menstruation is something to hide and not to speak about, associated with shame [4, 5]. This stigma and taboo surrounding menstruation translate into stress, shame, and fear of leaking or staining. Furthermore, concealment of menstruation has a negative impact on the correct diagnosis and treatment of problems by health professionals [12, 13] and contributes to the lack of solutions to associated issues like clinical diagnosis, lack of information, or the affordability of menstrual hygiene products (MHPs) [2, 14–17].

In Spain, in May 2022 a draft reform of the Organic Law 2/2010 on sexual and reproductive health and voluntary interruption of pregnancy was approved. It includes the right to medical leave due to painful menses and menstrual disorders for the first time [18]. In September 2022, the sales tax on MHPs was reduced from 10 to 4%, finally considering MHPs essential goods by law [19]. Despite these milestones and the efforts of social movements to promote menstrual health in recent years, the state of menstrual education in Spain has not been addressed.

In Spain, there is no part of the educational curriculum specifically dedicated to menstrual health. While menstruation is mentioned for its role in reproduction, it is not explicitly specified to teach menstrual health. Therefore, it is up to individual teachers' discretion how to address the topic and how much to cover. A similar situation is described by Curry et al. about the education curriculum in Australia [9]. The analysis of how educational laws have included sex education in Spain allows us to conclude that from the space of transversality opened by the LOGSE in 1990 to the last regulation of the LOMLOE (2020), educational laws allow, but do not guarantee, sex education. The inclusion of contents on sexuality within the specific subjects of the curriculum has not guaranteed the systematic and rigorous training of students throughout Spain.

The educational curriculum, established in the Spanish State official newsletter (BOE), includes menstruation’s role in reproduction but does not address menstrual health [20]. However, while still following the directions given in the national curriculum, regional governments do have the power to give more attention to menstrual health beyond reproduction. Thus, the content on menstrual education can differ from one region to another. There are a few regions whose regulations do not specifically mention menstrual health but allude to sexual education and sexual hygiene, which might/might not include menstrual health (e.g., Comunidad Valenciana [21], Castilla y León [22]); in others, the legislation is more general and the closest topic to menstruation is human reproduction (e.g., Castilla la Mancha [23], Comunidad de Madrid [24]). For more exhaustive information on menstrual education in the different regions, see supplementary material.

Increasing formal education on menstrual health, and to what extent, is left in the hands of individual instructors of the subject Biology and Geology. Family members have often been expected to provide menstrual health information informally to bridge this gap. Thus, menstrual literacy can come from both formal (school) and informal (friends and family) routes which can contribute to misinformation and perpetuate myths [25].

Although there is anecdotal evidence that menstrual literacy in Spain is low, there is no robust data available, hindering the ability to advocate for policies and regulations to address the issue. This study’s aim is an effort to understand how Spanish people, both those who do and do not menstruate, learn about menstruation formally and informally, and how this knowledge has impacted their emotions, particularly at menarche. Based on the recommendations provided by survey respondents, we also identify key areas for action so that all PWM in Spain are able to achieve “complete physical, mental, and emotional wellbeing” throughout their cycle [4, 14].

## Materials and methods

A survey approach, containing both quantitative and qualitative items, was deemed appropriate due to the exploratory nature of the research. Following the guidelines of the Research Ethics Committee of the Polytechnic University of Valencia (approval number P01_24_03_2022, 29 April 2022), survey participants were informed of the data processing regulations and had to consent to all the information provided at the beginning of the survey. The survey was conducted between May 2021 and January 2022.

### Questionnaire

The questionnaire was informed by previous studies [14–16, 26] to obtain qualitative and quantitative information on how menstruation is perceived and experienced in Spain. Data was gathered through single and multiple choice, rating scales, dropdown, and open-ended questions by using the digital platform Typeform [27] (the original questionnaire was in Spanish; an English translation is included as Supplementary Material). The questionnaire consisted of 43 questions addressing menstrual material choice, level of comfort when talking about menstruation, the social impact of menstruation, feelings experienced, information sources used to learn about menstruation, and information received before menarche. Some questions are intended for PWM only, others for people who do not, and do not expect to, menstruate (PWDNM), and most are common to both groups. The questionnaire took an average of 17 minutes to complete.

The questions can be classified into four sections: Demographic information, Knowledge about menstruation, Information sources, and Perception/Feelings. In the first section, quantitative variables, such as age (keyboard entry), or qualitative variables (dropdown options), such as region, country, gender, menstrual phase, or educational level, were included. Demographic and gender information was requested to contextualize the data, but no personal information that would potentially identify the respondent was collected.

In the second section, quantitative variables such as knowledge of what the first bleeding was or the knowledge on how to handle it, were investigated. For these variables, numerical scales from 1 to 10 were employed, where 1 meant “I had no information” and 10 “I was completely sure what it was or how to manage menstruation”. Qualitative variables such as what menstrual hygiene products were known at menarche or what information was provided at school, are included using multiple option answers. For example, for the question about what information was provided at the school, the answer offered different options: nothing, only biological aspects, some types of PHM, information on how to manage menstruation or even cultural aspects.

In the third section, information sources, qualitative variables, such as what type of primary information source was used during menarche or what information sources have been used in the last five years were presented, via multiple option questions.

Finally, in the section on perception/feelings, qualitative and quantitative variables were presented to collect information on how normalized they believe the topic of menstruation is in Spain (scale from 1 to 10), how comfortable they feel talking about menstruation (scale from 1 to 10) and the feelings experienced during menarche (multiple option answer).

In addition, an open-ended question was presented for respondents to express comments or opinions on menstruation. The content validity of the questionnaire was initially tested on a sample of 45 people (both PWM and PWDNM) of different ages and backgrounds residing in Spain. Feedback was provided by experienced social science and health researchers, who helped refine the wording and assess the suitability of the questions. Data from the pilot study were not included in the final data set due to adjustments in the survey. The reliability and consistency of the questionnaire were checked using Cronbach's alpha on the questions suitable for evaluation, which provided a value of 0.81.

The survey was disseminated on social media: Facebook, Instagram, and Twitter. Selected social media accounts with a high number of followers, including a wide variety of topics -scientific communicators, public personalities, influencers, activists, teachers, etc.- were approached by the first author and asked to share. In addition, it was shared through WhatsApp groups, networks, and face-to-face conversations, with the goal of the survey being as representative of the Spanish population as possible.

The research was open to people over 14 years old who were either born in Spain or a Spanish territory (and resided anywhere in the world) or were born abroad and currently lived in Spain or a Spanish territory. The survey was open to both biological sexes (participants were not asked to identify their gender), although some questions were different depending on whether respondents had experienced menses.

## Results

### Sample

The survey was completed by 4028 respondents, 513 were PWDNM (12.7%), and 3515 were PWM (87.3%). Respondents were classified as PWM—currently menstruating (3250—92.5% of the female group), PWM—experiencing menopause, (242—6.9% of the female group), and PWM—pre-menarche (23—0.7% of the female group) (Table 1).

**Table 1:** Sample statistics summary

Variable	Frequency	Percentage
People who don’t menstruate	513	12.7%
PWM currently menstruating	3245	80.7%
PWM experiencing menopause	242	6.0%
PWM pre-menarche	23	0.006%
**Decade of Birth**		
40 s (1940—1949)	8	0.2%
50 s (1950—1959)	49	1.2%
60 s (1960—1969)	176	4.4%
70 s (1970—1979)	679	16.9%
80 s (1980—1989)	1255	31.2%
90 s (1990—1999)	1479	36.7%
00 s (2000—2009)	382	9.5%
**Region at time of survey**		
Andalucía	466	11.6%
Aragón	61	1.5%
Principado de Asturias	95	2.4%
Islas Baleares	43	1.1%
Canarias	146	3.6%
Cantabria	47	1.2%
Castilla la Mancha	138	3.4%
Castilla y León	229	5.7%
Cataluña	456	11.4%
Comunidad Valenciana	833	20.8%
Extremadura	55	1.4%
Galicia	250	6.2%
La Rioja	25	0.6%
Comunidad de Madrid	782	19.5%
Región de Murcia	79	2.0%
Navarra	41	1.0%
País Vasco	186	4.6%
Ceuta y Melilla	2	0.0%
Out of Spain	79	2.0%

Respondents varied from 14 to 80 years old (born in 1941), although 73% of respondents were aged between 22 to 43 years. The average age of respondents was 34.9 with a standard deviation of 10.4.

The respondents that hold a university degree amounts to 71%. It is thus likely that the results of the study do not adequately represent lower socio-economic groups.

### Data analysis

Data was collected responding to demographic details as well as 10 quantitative content questions and one qualitative content question. Descriptive and inferential statistical analyses were performed with SPSS software. Shapiro–Wilk test of normality determined that many questionnaire scores were not normally distributed. When the normality assumption was not met, nonparametric statistical methods were employed, such as independent samples Kruskal–Wallis test, and the Mann–Whitney U test. The chi-square test was used to analyse the relationships between qualitative variables (*Feelings* versus *Source of information*), while the existence of a dependency relationship between numeric versus descriptive variables (*Information on what the first bleeding was* vs *Feelings* and *Information on how to manage menstruation* vs *Feelings*) was studied using the Student t-test and the normal distribution. Linear regression was employed to study the relationship between frequencies of feelings and perceived information on menstruation.

The last question of the survey was’Please use this space to share any doubts, comments, or reflections on this topic. You can also share experiences or anecdotes related to menstruation…’ and 1165 respondents answered this question. Responses to this question were openly coded in Nvivo 12 to thematically analyse menstrual health topics which respondents felt were important to discuss when given an unrestricted opportunity. Codes which are relevant to menstrual literacy are provided in Table 2. Responses assigned these codes were interrogated to interpret the quantitative results presented here [28].

**Table 2:** Codes relevant to this manuscript

Code	Meaning
Desired Information	Information respondents wished to have or to have learned about earlier
Early Menarche	Mention of early menarche experience (i.e., before learning about menstruation at all)
Education	Mention of education about menstruation
Educating all	Mention of menstrual education addressing the whole classroom, not only PWM
Education at school	Comments on the importance of menstrual education being addressed at school
Lack of education	Mention of lack of menstrual literacy
Informal education	Mention of information received from informal sources e.g., mothers, friends, internet
Emotional dimension	Mention of menstruation’s impact on mood
Emotional discredit	Ideas, reactions or moods blamed on menstruation
Menarche experience	Experiences, anecdotes, and comments on the experience of menarche
Men's knowledge	Mention of men's knowledge or lack of about menstruation (coded as men rather than PWDNM as this is how it was articulated in the data)
Men’s view	Comments from men about their perception of menstruation or the perception of their male friends (coded as men rather than PWDNM as this is how it was articulated in the data)
Menopause	Mention of menopause
Pain	Mention of pain related to menstruation
Questions	Questions about menstruation

### Knowledge about menstruation at menarche

To the question ‘*When you had your first period, how much information did you have regarding what the bleeding could be’? being 1 ‘No idea of what was going on’, and 10 ‘I knew exactly what it was’*, 35.7% of respondents who menstruate or are experiencing menopause scored 5 or less indicating that a significant proportion of PWM did not remember properly understanding why they were bleeding.

**Fig. 1:**
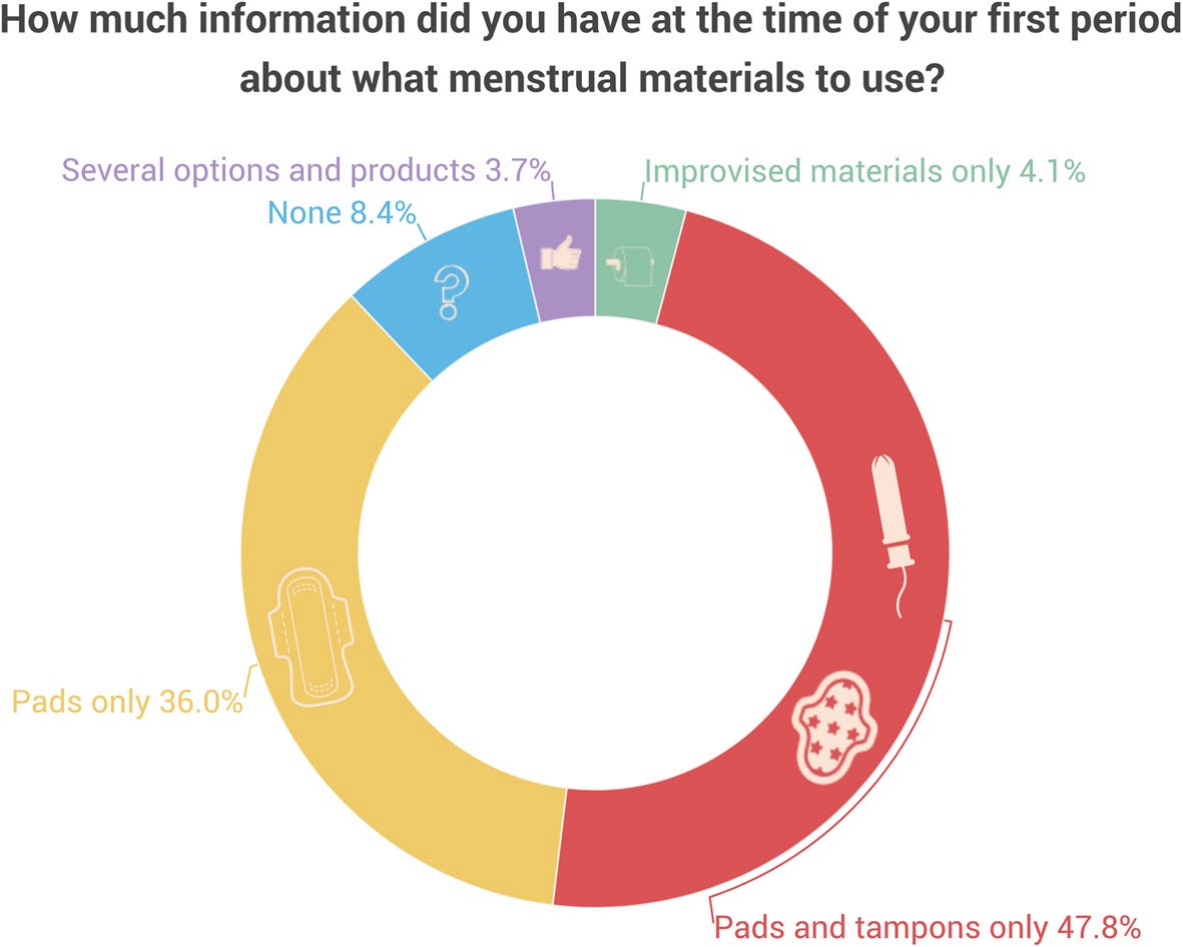
Knowledge of different types of menstrual materials at menarche

Figure 1 shows what materials respondents who menstruate or are experiencing menopause knew how to manage the bleeding at the time of their first period.

**Fig. 2:**
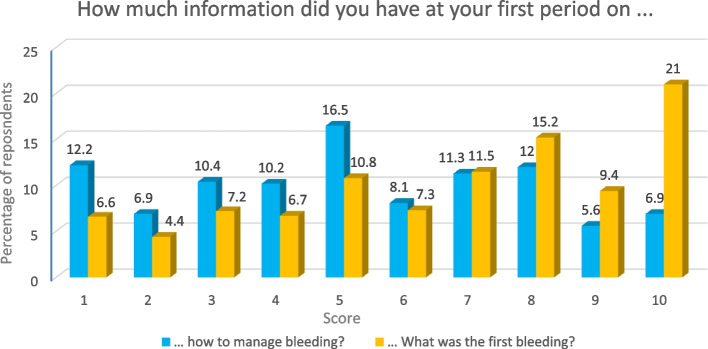
Knowledge about menstruation at menarche. Respondents included people who have reached menarche

When asked *‘When you had your first period, how much information did you have on how to manage the bleeding?*' from 1 to 10, 1 being *'None'*, 5 ‘*Enough’*, and 10 '*A lot of information*’, 56.1% of respondents who menstruate or are experiencing menopause scored 5 or less (Fig. 2). A low average was obtained for the knowledge on what the bleeding was (Mean = 6.58, Median = 7, Mode = 10, Std. Deviation = 2.23), with a significantly lower mean for information on how to manage the bleeding (Mean = 5.23, Median = 5, Mode = 5, Std. Deviation = 2.69) (Fig. 2).

To determine whether these perceptions of knowledge have changed over time, we considered respondents’ decade of birth (1950s-2000s). There has been a small, but statistically insignificant, increase in terms of the perceptions of people who currently or previously menstruate/d and the information they perceived they possessed prior to menarche, regarding what bleeding was and how to manage bleeding.

### Sources of information

#### Main source of menstrual management information during menarche

**Fig. 3:**
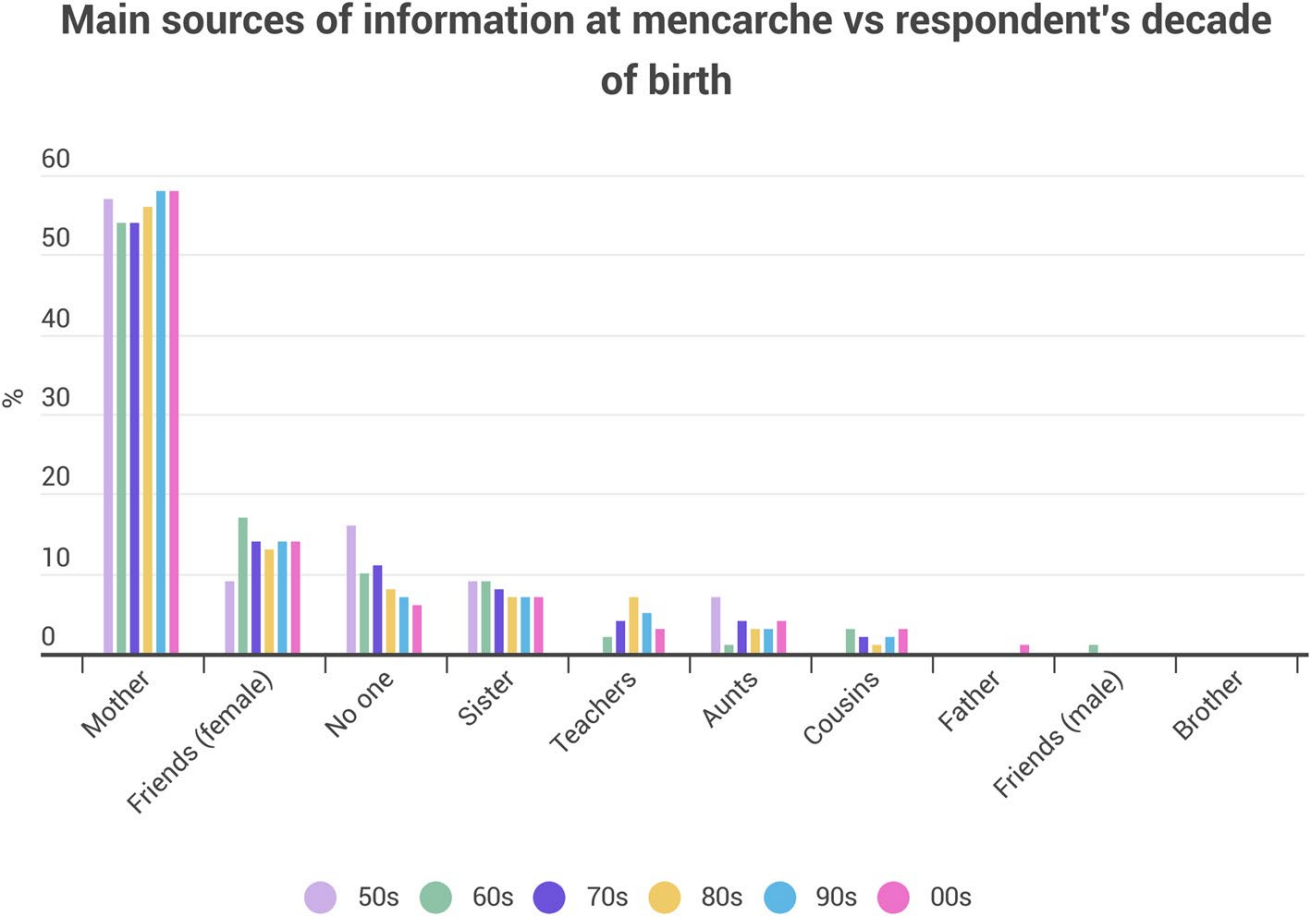
The main sources of information at the time of menarche compared to the decade in which the PWM was born. Only those who had reached menarche were asked this question. Multiple selections were allowed

Mothers were the most common source of practical information at the time of the first period (72.6%) while 10.7% of respondents who menstruate or are experiencing menopause learned how to use MHPs by trial or by following instructions given in MHP packaging, but without the support of anybody else (Fig. 3).

When comparing the rates of “mother”, “female friends”, and “sister” as sources of information on menstrual management to respondents’ decades of birth, no significant differences were found (*p*-values = 0.416, 0.412, 0.420, respectively). However, the incidence of not consulting anyone has decreased over time (linear regression; incidence = -0.0017*decade + 3.5137, R^2^ = 0.78 and *p*-values = 0.655). Nevertheless, approximately 7% of PWM born in the 2000s who have reached menarche still did not consult a source of practical guidance at menarche (Fig. 3).

#### Sources of information consulted in the last five years

**Fig. 4:**
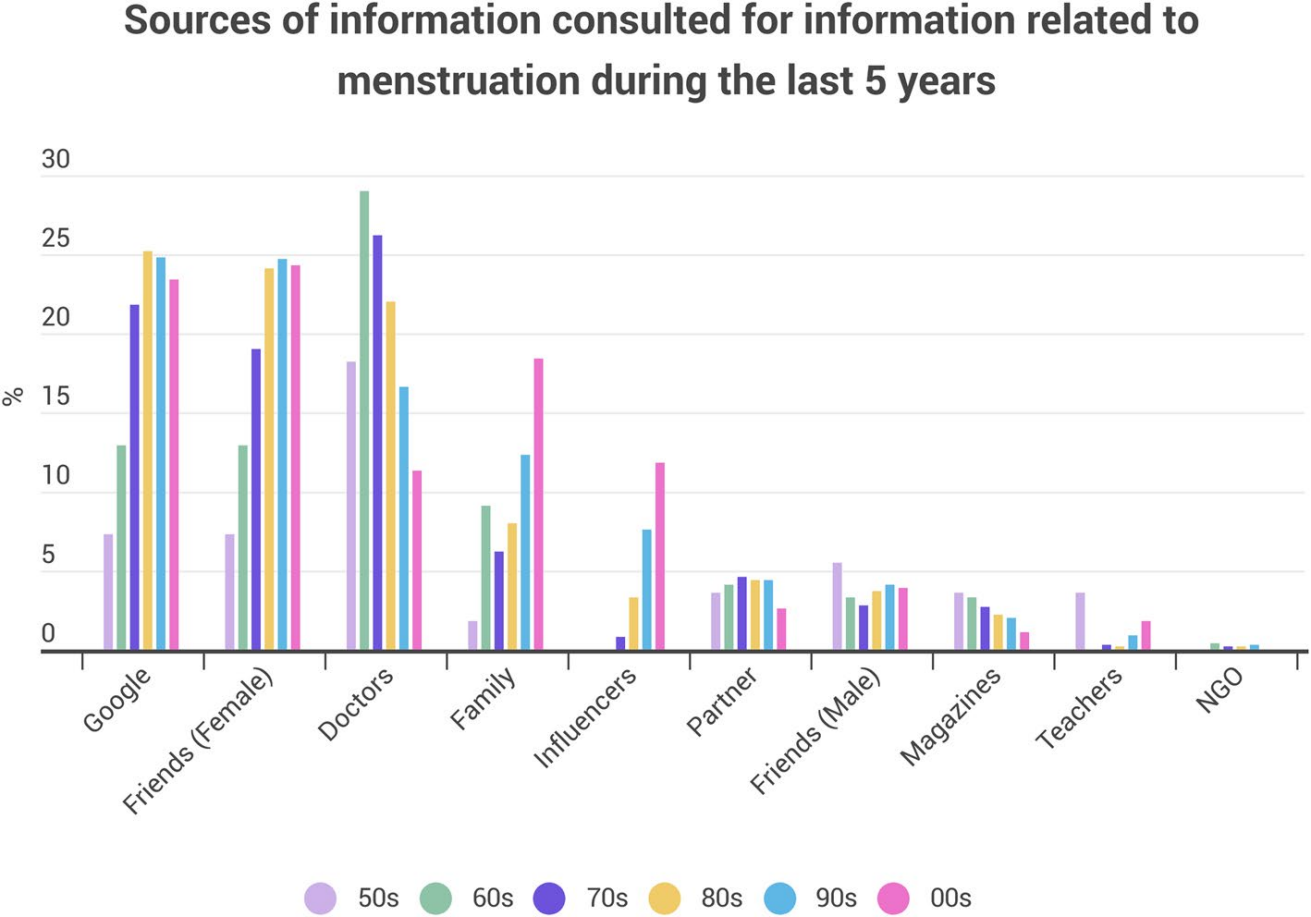
Sources of information on menstruation consulted in the past five years, by the decade of birth of the respondent. Every participant was asked this question. Multiple selections were allowed. NGO: Non-Governmental organisation / Non-profit organisation

Every respondent was asked what sources of information they have consulted in the last five years regarding menstruation—either looking for information for themselves or someone else (Fig. 4).

The main source of information consulted in the last five years has been Google (19%), but there is an increasing linear trend with the decrease of respondents’ age, from 7.3%, selected by people born in the 1950s, to 25%, selected by people born in the 1980s (Incidence = 0.0034*decade—6.5662; R^2^ = 0.75, *p*-value = 0.026). For those born in the 1990s and 2000s there is a slight decrease, which may be due to the use of social media. Female friends stand as a second source of information in the last five years, with an increasing linear trend with decreasing age (Incidence = 0.0036*decade—6.8964; R^2^ = 0.86 and *p*-values = 0.001). Doctors represent the third most selected source of information with an average rate of 21%, decreasing with decreasing age of respondents (Incidence = -0.0022*decade + 4.4826; R^2^ = 0.39, *p*- value = 0.025). Looking for information within the family is positioned as the fourth source of information, and also shows an increasing linear trend (Incidence = 0.0027*decade—5.2126; R^2^ = 0.8, *p*-value = 0.01) over the decades, reaching 18.4% for respondents born in the 2000s. Influencers occupy the fifth position, with a significant linear trend (Incidence = 0.0024*decade—4.729; *p*-value = 0.008, R^2^ = 0.87), decreasing with participants’ age and reaching 11.8% of the responses from those born in the 2000s, the same value as for doctors in this age group.

#### Education at school

When asked about what aspects of menstruation participants (all respondents) remember having learned at school, 74% answered the biological aspects of menstruation as part of reproduction, 15% about existing menstrual materials (including both MHPs and improvised materials), 5% how to manage the bleeding (e.g., how to use menstrual materials, how often to change them), and 2% cultural aspects associated with menstruation.

The timing of providing information about menses was highlighted as an important aspect to consider by several respondents who received the information after their first period. This quote illustrates the experience of many across the generations surveyed: “*When I had my first period (9 years old) I had no information whatsoever because when talks were given at my school about periods, pads, tampons, *etc*. I had already been on my period for two years…so the talk came a little late. I think this information should be given much earlier in schools because girls who have it the first ones feel very lost, ashamed, and alone”.* (PWM currently menstruating, E1576, Cataluña, YOB 1986).

#### Educating everyone

Participants often reflected on the need for menstrual literacy to include both PWM and PWDNM (articulated in the data as boys and girls). Some also shared experiences where adults who do not menstruate had a limited understanding of menstruation. Their answers (representative responses provided) suggested that the participants understood education targeting the whole classroom as a solution to knowledge deficits:

- 1. the lack of information boys receive leaving them without a basic understanding of periods;
  *In sixth grade (11–12 years old), the girls in my class started to have it (menstruation), and I asked why they went to the bathroom so much. Of course, since it was an uncomfortable subject, I looked like a bad person and I went home as such and without the answer*. (PWDNM, P396, Salamanca, Year Of Birth (YOB) 2000)
- 2. the normalisation of menstruation to prevent bullying and shaming in school and as adults;
  *We should inculcate it from an early age, both for girls and boys… so that if girls stain their pants, they will not be teased. *(PWM currently menstruating, P160, Aragón, YOB 1999)
- 3. preventing adult men from holding misconceptions or having no knowledge about menstruation.

*My boyfriend is 36 years old and I had to explain to him what a period was and where that blood was coming from. Another friend (who does not menstruate) thought that sanitary pads were for pee because we leak sometimes. I don’t understand how guys that old still don’t know anything about it. And let’s not get into the comments like, what’s wrong with you today? Are you having your period? They kill me.* (PWM currently menstruating, P637, Andalucía, YOB 1981)

*We men have little information about how disabling a painful period can be or whether it really influences the emotional state of women. There are a lot of rumours and stereotypes. In my case, my knowledge is limited to the women I have been able to ask directly and their experiences are very diverse. I would like to see quality outreach on those aspects to understand it (menstruation) better. *(PWDNM, P2786, Madrid, YOB 1965).

### Desired information

Open text was used by participants to share questions or information they deemed important to be available for everyone. Below, some quotes illustrate the topics that were commented on more frequently. These included information about endometriosis and other common menstruation disorders, a better understanding of what to expect from periods in terms of amount and consistency of bleeding, pre-menstrual symptoms, etc., that allows PWM to identify what is ‘normal’ and what is a reason to seek advice from a health specialist; if pain is a normal symptom of menstrual cycle, and a sort of reference to understand when it indicates that something is not right. More knowledge was desired on how the entire menstrual cycle affects the body, how hormones affect emotions, and how all these aspects change along their life span including menopause. A large proportion of the open text comments included a desire for the availability – not just in formal school settings—of more information focused on menstrual health issues and medical support.

*I would really like to know more about the cycles and how to take care of myself so that it doesn't hurt so much but there is very scattered information and it is difficult for me to get a good idea of how to organize and take care of myself.* (PWM currently menstruating, P174, La Rioja, YOB 1981)

*I think there is a lot of misinformation about the amount of bleeding that is normal. I have the feeling that many of us women have experienced brutal bleeding episodes (in my case, even anaemia) and we didn't know if it was normal or not and how to measure it.* (P2307, Galicia, YOB 1996)

*Very little information is provided about when we enter the phase of imbalances at a certain age. We suffer many hormonal alterations that we do not understand and think are not normal*. (PWM currently menstruating, P2998, Murcia, YOB 1976)

*How can I distinguish abnormal menstruation from diseases that look similar?* (PWM currently menstruating, P1772, Andalucía, YOB 1993)

*I think that there should be much more divulgation of issues such as endometriosis in young people since many times there are people who have a lot of menstrual pain that incapacitate them for their daily life and other people do not know the real pain they are going through*. (PWM currently menstruating, P382, Comunidad Valenciana, YOB 1995)

*I have read on the internet that feeling pain is not natural and should not happen, but many menstruating people in my environment feel pain. To what extent is it true that it should not hurt? Should I be worried?* (PWM currently menstruating, P3032, Cataluña, YOB 1998)

*I have always been struck by the concept of premenstrual syndrome on a psychic level, but I have never understood to what extent it is physiological*. (PWM currently menstruating, P3296, Madrid, YOB 1999)

### Feelings

The emotions experienced during menarche were included in the study to understand how knowledge about periods might affect mental health. Emotions are complex and can be experienced simultaneously. Thus, the answer to the question *‘What feelings did you experience when you had your first period?’* was asked to respondents who have reached menarche and allowed multiple choice and the possibility to add others.

**Fig. 5:**
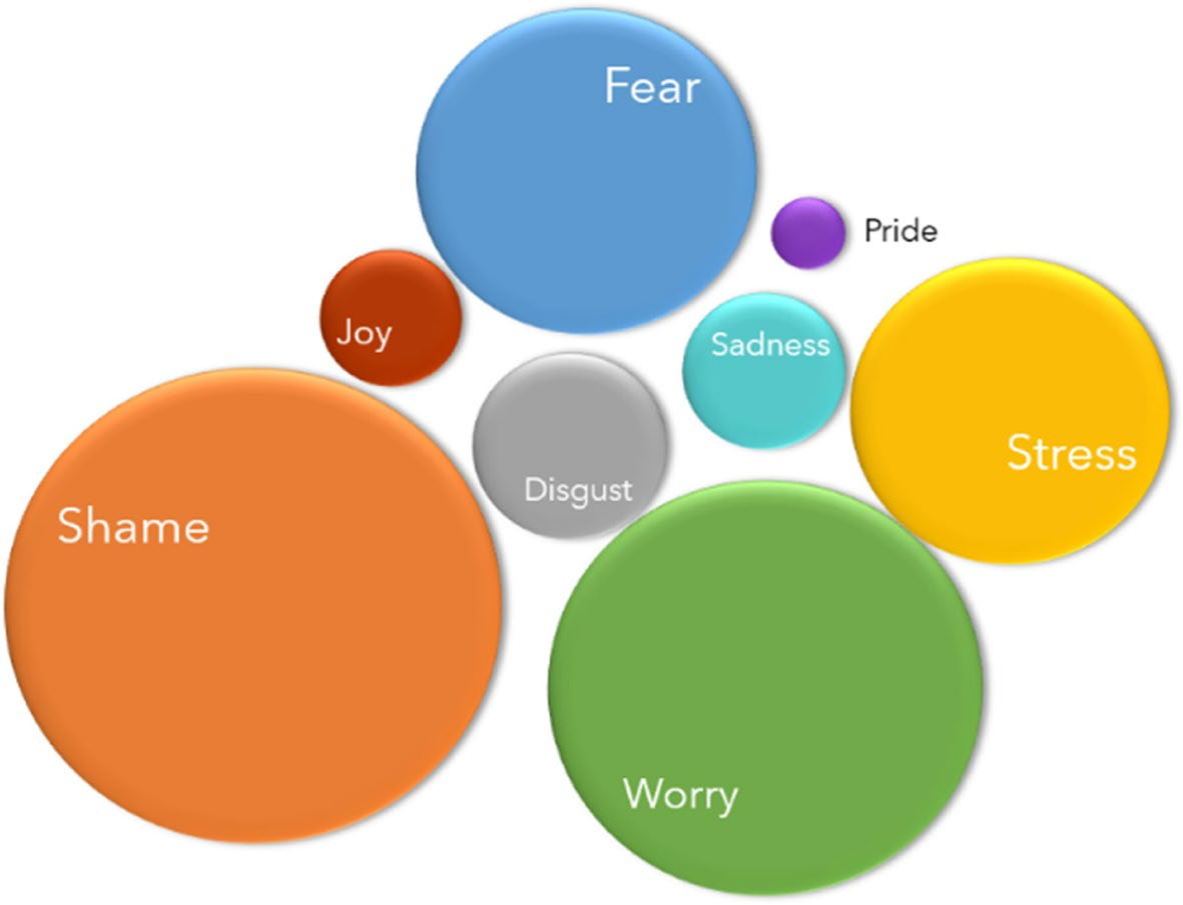
Feelings experienced during menarche. Size is proportional to the number of times that each emotion was reported. Bigger circles represent emotions that were reported more frequently than those in small circles

Figure 5 illustrates how frequently each emotion was experienced. The four most common emotions were *Shame* (23%), *Worry* (20%), *Fear* (16%), and *Stress* (15%). Significantly, these feelings are negative emotions.

The frequency of reported feelings was compared to the respondents’ decade of birth and analysed to study if emotions at menarche have changed over time. The results show no significant change (Table 3).

**Table 3:** Frequency of the reported emotions experienced at menarche by the respondents’ decade of birth

Decade of Birth	Percentages (%)								
	Joy*	Shame^**^	Fear^**^	Stress*	Pride*	Worry^**^	Sadness*	Disgust^**^	**Frequency**
1950s	16.3	8.2	8.2	6.1	4.1	18.4	0	6.32	49
1960s	7.4	28.4	13.6	15.3	6.8	20.5	2.3	6.3	176
1970s	9.6	32.5	14.4	16.6	4.3	24.7	10.8	10	679
1980s	9.6	36.2	20.6	19.9	5.1	27.6	11.5	11.6	1255
1990s	9.7	36.2	30.3	26.4	5.3	35.3	12.8	16.3	1479
2000s	9.4	33.8	32.7	29.6	5.2	37.4	13.1	20.7	382
Average	9.1	33.4	22.3	21.5	5.0	29.1	10.0	12.9	
Std. Dev	1.0	3.2	8.8	6.2	0.9	7.1	4.5	5.6	
Total	385	1394	959	898	206	1226	461	545	4028

Emotions with * showed a correlation with the information about what the bleeding was, and emotions with ** showed a correlation with the information on how to manage menstruation information

### Information vs feelings

**Fig. 6:**
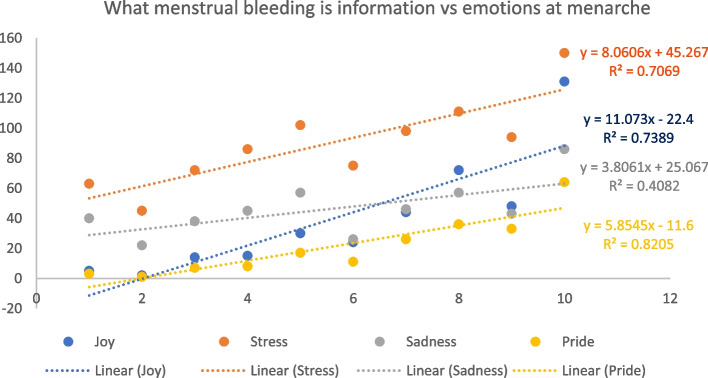
Linear regression between the perceived amount of information on what menstrual bleeding is and the feelings experienced at menarche

**Fig. 7:**
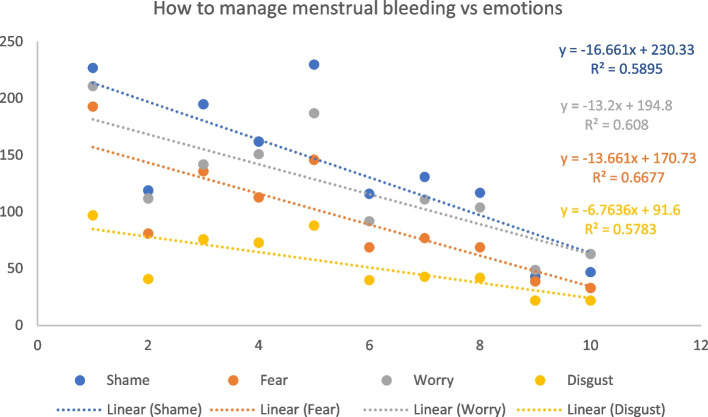
Linear regression between the information on how to manage menstrual bleeding and the feelings experienced at menarche

We compared the amount of information respondents who menstruate or are experiencing menopause perceived they possessed during their first bleeding to the emotions they experienced on menarche, to investigate any correlations (Table 3). Where there was a correlation, analysis was then performed to investigate whether there is a linear relationship between the frequency of each emotion experienced and the perceived information received on both what bleeding is and how to manage it. There is a positive trend between the frequency of *Joy*, *Pride*, *Stress*, and *Sadness*, as knowledge of what bleeding is increases (Fig. 6), suggesting that both positive and negative emotions increase with knowledge of what menstrual bleeding is. There is a negative trend between the frequency of *Shame, Fear, Disgust* and *Worry*, suggesting negative emotions decrease as knowledge of how to manage bleeding increases (Fig. 7).

## Discussion

This study is an effort to understand how menstruation is perceived and experienced, study menstrual literacy in Spain, and assess whether actions should be placed to promote menstrual literacy.

### Menstrual literacy at menarche

Participants often reported lacking sufficient knowledge about menstruation, including the biological aspects, existing MHPs, and how to physically manage periods, in line with studies in other high-income countries [2, 5, 10, 11, 29, 30].

Most respondents who have reached menarche perceived that they had limited information about menstruation before their first period and believe they could have been better prepared. There was no significant difference between the information on bleeding and menstrual management they reported having at menarche by respondents born between 1950–2010. This suggests that menstrual education may not have been updated, or at least kept pace, with the social, educational and political improvements that have occurred in Spain in the last 50 years.

School-based education is suggested by several researchers as a tool to address low menstrual literacy [9, 31]. Schools are useful sites for the dissemination of knowledge about menstrual health as they are considered a trusted source of information [9]. Education provided at school is an opportunity towards equity; ensuring that all schooled children have reliable basic information independent of their personal circumstances, e.g., economic status, level of education, presence of women in the family. In this study, a lack of information about menstrual disorders, what to expect from periods in terms of amount and consistency of bleeding, pre-menstrual symptoms, pain and knowledge of indicators that may suggest a medical issue or disorderhas been found. More knowledge on how the entire menstrual cycle affects the body is demanded by respondents to improve their menstrual health.

Another key aspect to consider is educational timing. The average age for menarche in our population was 12.08 years, in line with the range provided by previous studies (11.9—12.1 years) [32]. Older age at menarche is associated with more information [33], probably because those who experience menarche later have more time and chances to learn about it. In our study, several respondents who experienced early menarche commented on how they received the information too late and the negative impact this had on how they lived through their first period. Thus, education on menstruation needs to be provided before menarche to ensure girls are aware of what happens to their bodies, as suggested by several respondents, and previous studies [3, 14, 30, 34–36].

The presence of menstruation in Spanish schools’ curricula is mostly limited to its role in reproduction (i.e., biology), with less information provided on menstrual management. Focusing menstrual education on its biological aspects is a common approach, although incomplete; it does not address medical, social, and emotional dimensions [37] and disregards relevant discussions like how to identify irregularities in menstrual cycles or what to do when experiencing them [9]. Respondents who menstruate or are experiencing menopause indicated they would have appreciated having this knowledge at menarche and would prefer that PWDNM also had this knowledge.

Mandated school curricula need to clearly indicate what aspects of menstrual literacy must be covered to ensure teachers provide a comprehensive approach, rather than teachers being able to select for themselves where the focus should be [9]. A comprehensive education should address the biological cycle, all available menstrual materials and methods and how to use them and what to expect in terms of blood and potential discomfort to provide the ability to identify problematic symptoms in earlier stages [38], and tactics on how to manage menstrual pain. Additionally, emotional and social aspects of menstruation could be discussed, and efforts should be placed on normalising the conversation and creating a safe environment to ask questions to reduce the shame and stigma associated with menstruation [39].

In efforts to educate on menstruation, PWDNM should be educated too. Not limiting menstrual literacy education to PWM was repeatedly suggested by respondents, who see better and broader menstrual education as a means to reduce bullying, prevent misconceptions and increase the understanding of menstruation related matters and empathy. It is not possible to break social norms and taboos without addressing the entire population [36]. Furthermore, PWDNM are often responsible for mockery and shaming, and thus need to be part of the change. Consequently, mixed groups for menstrual education have been encouraged in the literature, although it is advised to reserve time for further discussion and openness in single-gender groups as they might feel more comfortable asking questions [9, 40].

### Menstrual literacy and menstrual disorders

Beyond a desire for greater menstrual literacy of PWM and PWDNM, respondents desired more information on pain management and potential menstrual disorders, e.g., how to prevent or treat pain and menstrual discomfort beyond contraceptives, a better understanding of the menstrual cycle and its changes during the lifespan, premenstrual syndrome, menopause, how to recognise what is ‘abnormal’ bleeding; how much pain is ‘normal’ during menstruation, and the need for more information on specific disorders, e.g., endometriosis. Education on menstrual disorders has been historically marginalised (see e.g., [41]); it would be useful to integrate such information when designing menstrual education strategies in Spain.

### Sources of menstrual management information at menarche

As respondents’ age decreased, the number indicating not having any source of practical information at menarche decreases. This indicates that fewer people go through menarche without any source of information. Still, 7% of PWM and have reached menarche born in the first decade of 2000s reported having no external source of information at menarche. This number, although considerably reduced compared to other decades, is not negligible. It would be interesting to study why this happens, if it is due to the shame of asking, lack of sources of information, or other causes.

The relevance of mothers as a primary source of information is clear, in line with previous studies [4]. Moreover, mothers' role as the main source of information remains predominant over time. Mothers, relatives, and friends are important for social support and practical guidance. The immediacy of this support, as well as the easy access to the information on the internet, are undeniable. Nevertheless, the reliability of the information is not being considered. The support offered by mothers or other people is limited to their own knowledge, and it can contain taboos and misconceptions [4]. The internet is a door to myriads of information as much as it can be a highway to misinformation, as noted elsewhere [42, 43].

Thus, there is a need for improved formalised education to provide reliable menstrual information whilst concurrently developing critical thinking skills [43]. Education provided in schools might help to ease the responsibility for mothers, uniparental families, families with two fathers, etc. Education at school will contribute to ensure reliable information for everyone independently of their personal circumstances.

### Information & feelings

Although some positive emotions associated with growing up were reported, menarche invoked negative emotional responses in most cases [14]. The amount of information that PWM possess during their first bleeding influences how they experience menarche [44]. This is consistent with several studies that found that participants felt underprepared and recalled high levels of fear and distress at menarche if they were unaware of menstruation [5, 10, 33].

Our results show that knowledge on the biological causes of periods increases the experience of both positive (*joy* or *pride*) and negative emotions (*stress* and *sadness*). Similarly, a study in Myanmar showed that girls who knew about the occurrence of periods and their links to reproduction did not have significantly less fear and reported higher levels of embarrassment [33]. A possible explanation for the increase in negative emotions is that the information girls received about periods already contributed to the narrative of shame and embarrassment [45–47]. Another possibility is that those who knew the cause of the bleeding were also aware of its long-term implications (e.g., years of managing periods, the potential for pregnancy). Reliable and positive information is needed to improve girls’ experience at menarche [33, 46]. Conversely, positive emotions experienced during menarche are usually related to the idea of growing up (i.e., pride) and understanding menses as a sign of ‘maturity’ or ‘becoming a woman’ (note this has been shown to contribute to gender dysphoria for PWM but do not identify as women or girls [4]). Knowledge on how to manage menstrual bleeding (e.g., how to use MHPs, how often to change MHPs) appears to significantly reduce the experience of negative emotions. In general, the increase in knowledge about managing menstrual bleeding reduced the negativity associated with menarche, as observed elsewhere [4].

Menstrual education before menarche might contribute to girls feeling more prepared and reducing negative emotions during menarche. However, this alone does not make shame or fear disappear, nor eliminate the taboo or social restrictions as menstrual experiences are shaped by sociocultural context [31] and the immediate environment of young people has a strong influence [33].

### Strengths and limitations

Despite the best efforts of the first author, the respondents of this study do not represent lower socioeconomic groups and vulnerable sub-populations; a frequent limitation in these case studies [4]. There is a high proportion of well-educated and high/middle-income respondents. It is expected that low-income populations and vulnerable subpopulations (homeless, immigrants, refugees, etc.) lack access to MHPs and probably have lower menstrual health literacy [48]. Limitations also include the inability to verify accounts.

Regardless, the findings make important contributions to the literature. First, we provide a large, empirical data set about menstrual literacy in Spain and its impacts on emotions at menarche. We have investigated how knowledge links to menstrual health. This research contributes to our understanding of the information that needs to be included in any intervention to increase menstrual health literacy, and the impact different types of information have on how menarche is experienced.

## Conclusions

Menstrual literacy should be addressed in Spain as many PHM lack sufficient information on how to manage menstruation when they experience it for the first time. In addition, PWDNM are ill-prepared to support PWM due to a lack of knowledge, particularly beyond the biological basis of menstruation to consider management and lived experiences.

The results of this study show the relevant role that information plays on how menstruation is experienced in Spain. Furthermore, information must not only be reliable and comprehensive but also be delivered early in life. Knowledge on how to manage menstruation decreases the experience of negative emotions (shame, fear, worry, and disgust) during menarche. Menstrual health education should be provided before menarche and should include the biological cycle, MHP and methods and how to use them, how to identify problematic symptoms in menses, tactics to manage menstrual pain, and not disregard the emotional and social aspects of menstruation.

This paper is a call to action to improve menstrual education in Spain. It is advised to include education about menstrual health in the curricula to guarantee every child is provided with reliable information independently of their personal circumstances.

## Supplementary Information

**Additional file 1**. Content of Menstrual Education Curriculum.

## Data Availability

All the data (used and analyzed) in this study are available from the corresponding author on request.

## Abbreviations

MHP: Menstrual hygiene products
PWM: People who menstruate
PWDNM: People who do not menstruate
