# Supplementary material for: Spanish menstrual literacy and experiences of menstruation
Source: BMC Womens Health. 2023 Apr 4;23:161. doi: 10.1186/s12905-023-02293-4 (PMC10074887; doi:10.1186/s12905-023-02293-4)
Supplement: Supplementary file 1 — Additional file 1. Content of Menstrual Education Curriculum. [file 12905_2023_2293_MOESM1_ESM.docx]

**Content of Menstrual Education Curriculum**

The analysis of how educational laws have included sex education in Spain allows us to conclude that from the space of transversality opened by the LOGSE in 1990 [1]to the last regulation of the LOMLOE (2020)[2], educational laws allow, but do not guarantee, sex education. The inclusion of contents on sexuality within the specific subjects of the curriculum did not guarantee the systematic and rigorous training of students throughout Spain.

Specifically, the content of the curricula of menstrual education in Spain can vary depending on the region, but in general it focuses on the following topics: 1. Menstrual cycle, 2. Body changes: physical and emotional. 3. Reproductive health: information on reproductive health, contraception, and sexually transmitted infections. 4. Healthy habits. 5. Hygiene. 8. Sexuality. 9. Social and cultural aspects of sexuality.

The following table shows the main educational contents taught about menstruation in the different Spanish regions.

| **Region** | **Content of Menstrual Education Curriculum** |
| --- | --- |
| *Regions that incorporate the menstrual cycle into their curriculum* | |
| Andalusia | Menstrual cycle. fertilization, pregnancy and childbirth. Analysis of the different contraceptive methods. Assisted reproduction techniques Sexually transmitted diseases. Prevention. The human sexual response. Sex and sexuality. Sexual health and hygiene.[3] |
| Catalonia | Topics include anatomy and physiology of female reproductive organs, reproductive health and development, menstrual cycle and its changes.[4] |
| Valencia | Topics include anatomy and physiology of female reproductive organs, reproductive health and development, menstrual cycle and its changes, contraception, and sexual harassment.[5] |
| Castilla y León | Topics include anatomy and physiology of female reproductive organs, reproductive health and development, menstrual cycle and its changes, contraception[6] |
| Castilla la Mancha | The reproductive function. Sexuality and reproduction. Physical and psychological changes in adolescence. Sexual health and sexual hygiene. Anatomy and physiology of the reproductive system. The menstrual cycle. Fertilization, pregnancy and childbirth. Analysis of the different contraceptive methods. The sexually transmitted diseases. Assisted reproduction techniques.[7] |
| Extremadura | The menstrual cycle. Fertilization, pregnancy and childbirth. Analysis of the different contraceptive methods. Sterility. Assisted reproduction techniques. Sexually transmitted diseases. The human sexual response.[8] |
| Asturias | The curriculum focuses on providing education on the physical and psychological changes associated with puberty, including menstruation, and how to manage them. It also provides education on how to take care of one’s reproductive health. [9] |
| Murcia | The menstrual cycle and its stages. Fertilization, pregnancy and childbirth. Analysis of the different contraceptive methods. Assisted reproduction techniques. Sexually transmitted diseases. Prevention.[10] |
| *Regions that include sex education but not menstruation education* | |
| Cantabria | The reproductive function and its biological relevance: anatomy and physiology. Affective-sexual education. The importance of responsible sexual practices. Prevention of sexually transmitted infections and unwanted pregnancies. Appropriate use of contraceptive methods. No education on menstruation. [11] |
| Madrid | Affective-sexual education from the perspective of equality among people and respect for sexual diversity. The importance of responsible sexual practices. Assertiveness and self-care.  Prevention of sexually transmitted infections and unwanted pregnancies. Appropriate use of contraceptive methods. No education on menstruation.[12] |
| Galicia | Reproductive physiology, contraception, pregnancy, sexual and reproductive health, gender equality, sexual harassment, and healthy relationships. No education on menstruation.[13] |
| Aragon | Includes activities that enhance the understanding of the biological and sociocultural aspects of sexuality, as well as information about hygiene, contraception, and gender roles. No education on menstruation.[14] |
| La Rioja | Reproductive physiology, contraception, sexual and reproductive health, gender equality, and healthy relationships. No education on sexuality or menstruation.[15] |
| *Regions that do not include sex education and menstruation education* | |
| Basque Country | The curriculum provides education on the physical and psychological changes associated with puberty. No education on sexuality or menstruation.[16] |
| Balearic Islands | Discusses the physical, psychological, and social aspects of sexuality. No education on sexuality or menstruation.[17] |
| Canary Islands | Affective-sexual education and recognition of sexual diversity. No education on sexuality or menstruation.[18] |
| Navarra | Affective-sexual education and recognition of sexual diversity. No education on sexuality or menstruation.[19] |

[1] Ministerio de Educación Cultura y Deporte, “Ley Orgánica 1/1990, de 3 de octubre, de Ordenación General del Sistema Educativo.,” *Boletín Of. del Estado*, vol. 238, pp. 28927–28942, 1990, [Online]. Available: https://www.boe.es/boe/dias/1990/10/04/pdfs/A28927-28942.pdf

[2] J. del Estado, “LOMLOE 3/2020, de 29 de diciembre,” *BOE núm.340*, pp. 122868–122953, 2020.

[3] Andalucía.Consejería de Educación., “Decreto 110/2016,” *Boletín Of. la Junta Andalucía*, no. 22, de 14 junio, pp. 15–16, 2016, [Online]. Available: https://www.juntadeandalucia.es/boja/2016/122/1

[4] Generalitat de Catalunya Departament d’Ensenyament, “Decret 187/2015,” *Dogc*, vol. Núm 6945, no. Disposicició, p. 305, 2015.

[5] Conselleria, “Decreto 87/2015, de 5 de junio, del Consell, por el que establece el currículo y desarrolla la ordenación general de la Educación Secundaria Obligatoria y del Bachillerato en la Comunitat Valenciana.,” *D. Of. la General. Valencia.*, pp. 1–28, 2020.

[6] E. Consejería, *Decreto 6/2013, de 31 de enero, por el que se modifica el Decreto 40/2007, de 3 de mayo, por el que se establece el currículo de la Educación Primaria en la Comunidad de Castilla y León*. 2013, pp. 8771–8786.

[7] C. y D. Consejería, Educación, “Decreto 40/2015 CLM, circulo de Educación Secundaria Obligatoria y Bachillerato en la Comunidad Autónoma de Castilla- La Mancha,” *D. Of. Castilla-La Mancha*, pp. 18872–20324, 2015.

[8] Decreto. Gobierno de Extremadura, “Decreto 98/2016, de 5 de julio, por el que se establecen la ordenación y el currículo de la Educación Secundaria Obligatoria y del Bachillerato para la Comunidad Autónoma de Extremadura,” *D. Of. Extrem.*, no. num 129, pp. 17347–18550, 2016, [Online]. Available: http://doe.gobex.es/pdfs/doe/2016/1290o/16040111.pdf

[9] P. de Asturias, “I . Principado de Asturias,” *Bopa*, pp. 1–3, 2018.

[10] C. Consejería de Educación, Cultura y Universidades, “Decreto n.^o^ 220/2015, de 2 de septiembre de 2015, por el que se establece el currículo de la Educación Secundaria Obligatoria en la Comunidad Autónoma de la Región de Murcia.,” *Boletín Of. la Región Murcia*, pp. 30729–31593, 2015, [Online]. Available: https://www.carm.es/web/pagina?IDCONTENIDO=51745&IDTIPO=100&RASTRO=c77$m4507,3993,21221

[11] C. D. E. Gobierno, “7.5.varios,” pp. 20441–21321, 2022.

[12] Vicepresidencia Consejería de Educación y Universidades, *Decreto 65/2022, de 20 de julio, del Consejo de Gobierno, por el que se establecen para la Comunidad de Madrid la ordenación y el currículo de la Educación Secundaria Obligatoria.*, no. 176, 26 de julio del 2022. 2022, pp. 396–716. [Online]. Available: https://www.bocm.es/boletin/CM_Orden_BOCM/2022/07/26/BOCM-20220726-2.PDF

[13] Xunta de Galicia, “DECRETO 156/2022, de 15 de septiembre, por el que se establecen la ordenación y el currículo de la educación secundaria obligatoria en la Comunidad Autónoma de Galicia,” *D. Of. Galicia*, 2022, [Online]. Available: https://www.xunta.gal/dog/Publicados/2022/20220926/AnuncioG0655-190922-0002_es.html

[14] C. y D. Departamento de Educación, “Orden de 9 de mayo de 2007, por la que se aprueba el currículo de la Educación secundaria obligatoria y se autoriza su aplicación en los centros docentes de la Comunidad Autónoma de Aragón,” *Boa*, 2007, [Online]. Available: http://benasque.aragob.es:443/cgi-bin/BRSCGI?CMD=VEROBJ&MLKOB=201255412828

[15] Consejería de Hacienda, “Boletón Oficial de la Rioja,” vol. 156, pp. 24921–24955, 2014, [Online]. Available: http://ias1.larioja.org/boletin/Bor_Boletin_visor_Servlet?referencia=1902409-1-PDF-486979

[16] Gobierno Vasco, “Currículo de educación básica,” *Currículo carácter orientador que Complet. el Anexo II del Decreto 236/2015*, pp. 1–695, 2015.

[17] C. de E. y Cultura, “Decreto 67/2008, de 6 de junio, por el cual se establece la orde- nación general de las enseñanzas de la educación infantil, la educación primaria y la educación secundaria obligatoria en las Islas Baleares,” pp. 47–54, 2008.

[18] MECD, “Proyecto de real decreto por el que se establece el currículu básico de la educación secundaria obligatoria y del bachillerato,” 2013.

[19] G. de Navarra, “DECRETO FORAL 71/2022, DE 29 DE JUNIO, POR EL QUE SE ESTABLECE EL CURRÍCULO DE LAS ENSEÑANZAS DE LA ETAPA DE EDUCACIÓN SECUNDARIA OBLIGATORIA EN LA COMUNIDAD FORAL DE NAVARRA,” 2015.
